# The niche matters: origin, function and fate of CNS-associated macrophages during health and disease

**DOI:** 10.1007/s00401-023-02676-9

**Published:** 2024-02-12

**Authors:** Adrià Dalmau Gasull, Martina Glavan, Sai K. Reddy Samawar, Kishan Kapupara, Joe Kelk, Marina Rubio, Stefano Fumagalli, Lydia Sorokin, Denis Vivien, Marco Prinz

**Affiliations:** 1 Institute of Neuropathology, Faculty of Medicine, University of Freiburg, Freiburg, Germany; 2 Normandie University, UNICAEN, INSERM UMR-S U1237, Physiopathology and Imaging of Neurological Disorders (PhIND), GIP Cyceron, Institut Blood and Brain @ Caen-Normandie (BB@C), 14000 Caen, France; 3 Department of Neuroscience, Yale School of Medicine, Yale University, New Haven, USA; 4 Institute of Physiological Chemistry and Pathobiochemistry and Cells in Motion Interfaculty Centre (CIMIC), University of Münster, Münster, Germany; 5 Laboratory of Stroke and Vascular Dysfunctions, Department of Acute Brain and Cardiovascular Injury, Istituto Di Ricerche Farmacologiche Mario Negri IRCCS, 20156 Milan, Italy; 6 Department of Clinical Research, Caen-Normandie University Hospital, CHU, Avenue de La Côte de Nacre, Caen, France; 7 Signalling Research Centres BIOSS and CIBSS-Centre for Integrative Biological Signalling Studies, University of Freiburg, Freiburg, Germany

**Keywords:** Macrophages, CNS, Development, Disease

## Abstract

There are several cellular and acellular structural barriers associated with the brain interfaces, which include the dura, the leptomeninges, the perivascular space and the choroid plexus epithelium. Each structure is enriched by distinct myeloid populations, which mainly originate from erythromyeloid precursors (EMP) in the embryonic yolk sac and seed the CNS during embryogenesis. However, depending on the precise microanatomical environment, resident myeloid cells differ in their marker profile, turnover and the extent to which they can be replenished by blood-derived cells. While some EMP-derived cells seed the parenchyma to become microglia, others engraft the meninges and become CNS-associated macrophages (CAMs), also referred to as border-associated macrophages (BAMs), e.g., leptomeningeal macrophages (MnMΦ). Recent data revealed that MnMΦ migrate into perivascular spaces postnatally where they differentiate into perivascular macrophages (PvMΦ). Under homeostatic conditions in pathogen-free mice, there is virtually no contribution of bone marrow-derived cells to MnMΦ and PvMΦ, but rather to macrophages of the choroid plexus and dura. In neuropathological conditions in which the blood–brain barrier is compromised, however, an influx of bone marrow-derived cells into the CNS can occur, potentially contributing to the pool of CNS myeloid cells. Simultaneously, resident CAMs may also proliferate and undergo transcriptional and proteomic changes, thereby, contributing to the disease outcome. Thus, both resident and infiltrating myeloid cells together act within their microenvironmental *niche*, but both populations play crucial roles in the overall disease course. Here, we summarize the current understanding of the sources and fates of resident CAMs in health and disease, and the role of the microenvironment in influencing their maintenance and function.

## Introduction

**Fig. 1:**
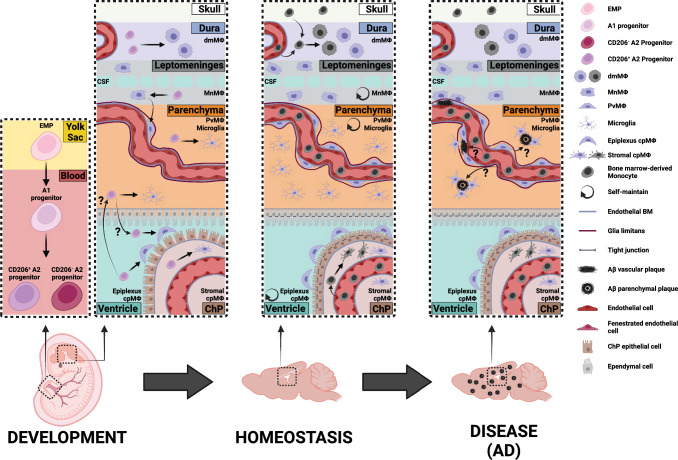
CNS resident macrophages during development, homeostasis, and Alzheimer’s disease (AD). During embryogenesis, erythromyeloid precursors (EMP) from the yolk sac differentiate to an intermediate immature population (A1) which transition into pre-macrophages progenitors (A2). These immature macrophages migrate through the developing blood vessels and start to invade the neural tube by E9.5. While some CD206^+^ A2 progenitors seed the parenchyma to become microglia, others engraft 1) the dura to become dural macrophages (dmMΦ), 2) the choroid plexus (ChP) to become epiplexus or stromal choroid plexus macrophages (cpMΦ), and 3) the leptomeninges to become leptomeningeal macrophages (MnMΦ). MnMΦ populate the perivascular spaces postnatally, differentiating into perivascular macrophages (PvMΦ). The PvMΦ are sandwiched between the endothelial basement membrane (BM) and the glia limitants. In the developed CNS, microglia, MnMΦ, PvMΦ, and epiplexus cpMΦ are long-lived and self-maintain. Instead, dmMΦ and stromal cpMΦ are slowly replaced by bone marrow-derived Monocytes. During AD context, not only microglia, but also PvMΦ and MnMΦ have a critical role in the clearance of vascular and parenchymal amyloid beta (Aβ) plaques. The maintenance of blood–brain barrier integrity, along with the possible contribution of monocyte-derived cells in clearing plaques, remains unclear

Central nervous system (CNS) resident macrophages are comprised of two main groups: parenchymal microglia and extra-parenchymal, CNS-associated macrophages (CAMs). Recent research has shown that CNS endogenous macrophages share a common origin, as both microglia and CAMs originate from embryonic yolk sac (YS) progenitors [34, 35] (Fig. 1). These precursors give rise to a pool of immature macrophages that migrate via blood vessels to colonize different regions of the developing CNS before the formation of the blood–brain barrier (BBB) [105]. In mice, CNS seeding of microglia occurs around embryonic day (E)9.5 [52], while in humans, the first microglia have been described at 4.5 weeks post-conception [70]. Upon entry into the CNS, the myeloid progenitor cells undergo extensive proliferation and differentiation within their respective *niches.* While microglial precursors seed the CNS parenchyma, CAM precursors seed CNS interfaces, giving rise first to leptomeningeal macrophages (MnMΦ) and choroid plexus macrophages (cpMΦ) [35]. In fact, recent data found that human microglia and CAMs are transcriptionally distinct already at 5 weeks post-conception [91]. Recent research in mice revealed that MnMΦ populate the perivascular spaces postnatally, subsequently differentiating into perivascular macrophages (PvMΦ) [61]. The leptomeningeal and perivascular *niches* differ in their cellular and extracellular matrix characteristics [40], thereby, imparting *niche*-specific signatures to the resident myeloid populations that include transcriptional profile, morphology, motility, self-maintenance capacity, and function [49, 53, 71, 98, 114]. Although microglial functions during homeostasis and disease have been long studied in depth [19, 82], the role of CAMs in physiological and disease conditions has just begun to be elucidated. So far, few studies have addressed this topic and have implicated CAMs in cerebrospinal fluid (CSF) flow dynamics [25], as well as in neurodegenerative [42, 95], cerebrovascular [77, 101], and neuroinflammatory diseases [22, 49, 78, 88, 90].

Besides their ontogeny, microglia and CAMs share some transcriptomic signatures including the expression of ionized calcium-binding adaptor molecule *(Iba)* 1, fractalkine receptor (*Cx3cr1*), and colony-stimulating factor (*Csf*) 1 receptor. Only the recent development of new technologies, such as single-cell RNA sequencing (scRNA-seq), has resulted in the identification of transcriptomic profiles that permit segregation of microglia and CAMs. For example, hexosaminidase subunit beta (*Hexb*), P2Y purinergic receptor (*P2ry) 12*, transmembrane protein (*Tmem) 119*, spalt-like transcription factor (*Sall*) *1*, sialic acid binding Ig-like lectin *(Siglec) h*, are enriched in microglia, whereas mannose receptor (*Mrc*) 1 (*or CD206*), membrane-spanning 4-domains subfamily A member (*Ms4a*)7, platelet factor (*Pf)4* are preferentially expressed by CAMs [35, 49, 126]. scRNA-seq has further revealed transcriptomic heterogeneity in CAMs located at different brain interfaces [114]. Specifically, MnMΦ were shown to be distinct from dural macrophages (dmMΦ) in the meninges. This is not surprising given that, despite collectively constituting the meninges that surround and protect the CNS, the leptomeninges and dura matter comprise distinct layers. The dura, positioned as the outermost layer, possesses unique characteristics such as high vascularity and abundance of collagen fibers, resembling more a connective tissue. Because the dura does not establish a BBB, it enables an exchange of blood-derived cells and molecules [4]. Consequently, these distinct *niches* are associated with unique CAM signatures. Similarly to meningeal macrophages, within the population of cpMΦ stromal- and epiplexus-located cells were shown to have distinct transcriptomic profiles. A recent study suggested the existence of more than one PvMΦ population, defined by presence or absence of CX3CR1 expression. A CX3CR1^neg^ population was identified, the function of which remains to be determined [101]. Importantly, comparison between human and mouse CAMs transcriptomic profiles highlighted the conservation of evolutionary markers in these cells [91].

The identification of differentially expressed genes in different myeloid populations has provided valuable knowledge for generation of novel mouse lines that specifically target microglia or CAMs [12, 50, 61–63]. The use of such innovative and promising mouse lines in models of neuroinflammation and/or neurodegeneration now provides the possibility of defining functions of specific CAM populations in brain homeostasis and pathologies and their potential as therapeutic targets.

## Origin and fate

It was long believed that microglia and CAMs constitute ontogenically distinct myeloid populations [44]. However, this concept was based on studies in bone marrow chimeric mice, where bone marrow-derived cells were detected in CNS perivascular spaces [8, 125]. These results were potentially due to the artificial disruption of the BBB and concomitant induction of aberrant chemoattractant signals in the CNS caused by whole-body irradiation [68]. The breakthrough came in 2016 when Goldmann et al., [35] employed fate-mapping mouse lines to demonstrate that CAMs and microglia both originate from the YS’s early erythromyeloid progenitors (EMPs). Differentiation into tissue-resident macrophages begins with a shift from EMPs to an intermediate immature population so-called A1, which then transitions into A2 pre-macrophages progenitors [34, 35, 52, 61, 113, 114] (Fig. 1). Based on *Mrc1* gene expression (which encodes CD206), two transcriptionally and phenotypically distinct A2 subpopulations were identified in the YS of E9.5 mice. As CD206 expression is limited to CAMs in the adult mouse CNS, this led to the assumption that CD206^neg^ A2 cells are microglia progenitors and CD206^+^ A2 cells are CAM progenitors [113]. However, this may not be the case, since tamoxifen induction in pregnant *Mrc1*^CreERT2/CreERT2^*R26*^tdTomato/tdTomato^ mice at E9.0 resulted in not only tdTomato^+^ CAMs but also tdTomato^+^ microglia in postnatal (P)14 brains [61]. This substantiates that microglia and CAMs share the same progenitor cells and, only once within their *niche*, do local factors determine their terminal differentiation.

To seed the developing brain at E9.5, progenitor populations travel within the bloodstream of the developing vasculature [105]. NCX1-deficient embryos, that lack a heartbeat and hence blood circulation, have normal numbers of YS pre-macrophage progenitors at E9.5 but lack microglia, indicating that blood circulation is required for seeding of the CNS [34]. The first A2 pre-macrophage progenitors are detectable in the mesenchyme surrounding the developing CNS at E9.0 and colonize the neural tube by E9.5 [60, 96]. Two recent studies have used *ex-vivo* time-lapse imaging on brain slices to investigate the CNS seeding of microglia progenitors, but with contradictory results. One study suggested that CD206^+^ A2 progenitors migrate from the mesenchyme first into the ventricle and subsequently into the developing brain parenchyma at E12.5. These cells then lose CD206 expression and acquire a microglia phenotype [41]. In contrast, the other study imaged slices of *Csf1r*-EGFP embryos and showed migration of Iba1^+^ cells from the developing parenchyma into the ventricle at E11, suggesting that these cells thereafter become cpMΦ [72]. These results indicate an opposite directionality at E11 compared to E12.5, which could be attributed to differences in cellular composition and thickness of the ventricle wall.

Once within their *niche*, microglia, MnMΦ, PvMΦ, and epiplexus cpMΦ are long-lived and self-maintain, thereby, retaining their YS origin, while dmMΦ and stromal cpMΦ are slowly replaced by circulating cells [35, 113, 114] (Fig. 1). These differences in population maintenance seem to be *niche*-dependent, since the dura mater and the choroid plexus stroma offer greater accessibility from the periphery compared to the other brain interfaces. In an attempt to further dissect the replacement of CAMs, Brioschi et al. [11] used a constitutive *Crybb1*-Cre line to label embryonic microglia and CAMs. As expected, microglia maintained their embryonic origin, but both MnMΦ and PvMΦ were suggested to be a mixture of embryonic and monocyte-derived populations that differed in CD38 and MHC2 expressions. Unfortunately, the authors did not address the turnover of cpMΦ or dmMΦ.

Importantly, during pathological conditions marked by physical damage to brain vasculature, there is a significant infiltration and active recruitment of peripheral monocytes [1, 22, 49, 71]. However, once the inflammation subsides, monocyte-derived cells usually not persist as residents in the CNS parenchyma [1, 49]. In contrast, it seems that methods depleting CNS myeloid cells create both accessible and available *niches* contributing to efficient monocyte engraftment and colonization [99, 121]. In a study where chronic partial microglia depletion was accomplished without disruption of the BBB, recruited cells were able to enter and engraft the CNS parenchyma while maintaining distinct transcriptional profiles from resident microglia [20]. Similarly, it has been suggested that in pathological states PvMΦ can also be replenished by the infiltration of circulating monocytes into perivascular spaces [59]. Interestingly, it has been proposed that dmMΦ turnover does not depend on blood circulating monocytes, but rather on engraftment from monocytes derived from calvarial bone marrow [21]. The latter topic is controversial as most of these studies are based on highly invasive imaging techniques that include skulls transplants or thinning of the skull bone, which may result in an inflamed condition. Recently, Sankowski et al. [91] assessed the engraftment of bone marrow-derived myeloid cells within the distinct compartments of the human CNS. Employing autopsies from female patients who had undergone sex-mismatched peripheral blood stem cell transplantation, revealing the presence of donor-derived Y + cells across all CNS interfaces, each displaying different engraftment rates. Remarkably, a time-dependent increase in engraftment was observed, with the highest exchange occurring in the choroid plexus, followed by the dura, and the slowest in the brain parenchyma—a pattern consistent with pre-clinical studies. Furthermore, transcriptional analysis unveiled a sustained activated profile of the engrafted myeloid cells. These findings suggest peripheral blood stem cell transplantation as a potential CAM replacement therapy for CAM-related disorders.

## Molecular cues for myeloid precursor recruitment to specific *niches*

PU.1 (or SPFI) is an essential transcription factor for the differentiation of macrophage progenitors into CNS resident cells. *PU.1*^*−/−*^ mice have diminished A1 and A2 progenitor populations and, consequently, no microglia or CAMs [35]. Another important transcription factor is interferon regulatory factor 8 (IRF8). Depletion of IRF8 in mice impairs A2, but not A1 progenitor cells, reducing the number of microglia and MnMΦ but not cpMΦ [52]. Neither the depletion of the chemokine receptor CX3CR1 nor of transcription factors such as Myb and Batf3 in late embryonic stages affect CNS resident macrophages numbers [35, 52]. Mice lacking C–C chemokine receptor 2 (CCR2), crucial for myeloid recruitment into the inflamed CNS [67, 85, 87], do not show any differences in microglia, MnMΦ and PvMΦ abundance, suggesting that CCR2 is not required for CNS seeding during embryogenesis. By contrast, *CCR2*^*−/−*^ adult mice showed a reduced number of cpMΦ, further supporting their continuous replenishment by peripheral monocytes [35].

It has been proposed that in the developing CNS, microglia entry into the ventricular and subventricular zones is mediated by CXCR4 interaction with CXCL12 expressed in the developing cortex [5]. Global deletion of *Cxcr4*, conditional deletion of its ligand *Cxcl12* in *Gfap*-expressing cells or injection of CXCR4 antagonist, reduced the amount of cortical microglia in mice [5, 112]. Accordingly, conditional deletion of *Cxcr4* in *Cx3cr1*-expressing cells at E10.5 reduced microglial cell numbers in ventricular and subventricular zones at E18.5 [112]. However, others have reported no expression of *Cxcr4* in microglia and normal microglia distributions in *Cxcl12*^*−/−*^ and *Cxcr4*^*−/−*^ embryos at E13.5 and E16.5, respectively [116]. This could be due to differences in embryonic time points or brain regions analyzed.

Little is known about the molecular mechanisms of CAM recruitment to their distinct *niches* during development. Recently, the migration of MnMΦ into the perivascular spaces early after birth was shown to be integrin-signaling dependent [61], suggesting a role for the unique extracellular matrix of the leptomeningeal and perivascular *niches* [39, 40]. Deletion of the gene encoding talin-1, *Tln1*, an important activator of β-integrin binding activity [13, 106], resulted in a normal distribution of microglia and MnMΦ in the embryonic CNS, but a 50% reduction of PvMΦ at P14 [61]. This implies an involvement of the extracellular matrix in MnMΦ migration into the perivascular *niche.* A functional role of mural cells was recently identified, which express several extracellular matrix molecules, since PvMΦ were highly reduced in *Notch3*^−/−^ animals that have defective maturation of arterial smooth muscle actin-positive cells [61]. The complex mutual interplay between perivascular cells, the extracellular matrix and associated factors, and the developing PvMΦ still requires elucidation.

## Vascular cues for erythromyeloid progenitor differentiation

Studies done in mice showed that a primitive, yet functional, BBB is formed by E15.5 and already contains some macrophage *niches*, structures where CAM differentiation proceeds to mature perivascular macrophages (PvMΦ), as discussed above. The full maturation of the perivascular space with arterioles containing smooth muscle cells and associated CAMs continues up to 2 weeks after birth, when PvMΦ start to appear [61]. The fact that other cellular components of the brain vascular barrier, like perivascular fibroblasts, appear between postnatal day P5 and P14 [48], suggests that the first two postnatal weeks are a critical period for extensive expansion of the cerebral capillaries [18].

The PvMΦ are sandwiched between glial and vascular basement membranes, a spatially restricted location which probably determines their limited motility and typical elongated shape, along the vessels. Spatial constraints could also affect the PvMΦ phenotype as previous in vitro studies have reported that forcing the elongation of cultured macrophages using narrow lines of substrate, such as fibronectin, upregulates arginase-1, Ym1 and the typical PvMΦ marker CD206 [64]. CD206 is a membrane-bound mannose receptor. Together with other glycans, including N-acetylglucosamine, glucose, fucose, sialic acid and heparan sulfate, mannose is present in the glycoproteins lining the luminal surface of blood vessels, contributing to the glycocalyx. Depending on vessel type, the glycocalyx has several physiological functions—it supports vascular tone and integrity, provides mechanotransduction signals, interacts with cytokines and growth factors, and regulates immune cell adhesion and rolling [69]. While this latter function has been studied in pathological contexts where immune cell recruitment supports inflammation [16], there are little data on CNS development. The glycocalyx appears as soon as blood flow is initiated in the embryo and is involved in normal vessel development [37, 43]. In this regard, the membrane glycoprotein neuropilin functions as a co-receptor for vascular endothelial growth factor receptor (VEGFR)2 and guides developmental angiogenesis [32]. The early formation of the glycocalyx could potentially affect the migration and recruitment of A2 pre-macrophage progenitors to the developing brain, an important step in the maturation of the cerebral vasculature [27].

Brain vessels are, therefore, key players in the ontogeny of CAMs, mediating the migration of the immature macrophages to the developing CNS and their placement in specific differentiation *niches.* Microglia seeding occurs around E9.5, when the BBB has not yet developed. In rodents, clusters of vascular endothelial-like cells are present in the developing CNS at E7.5–E8.5 [115] and start to organize into a branched vascular network by E9.5 [109], which correlates with microglia seeding of the CNS. Microglia exploit the forming vascular structures as paths to infiltrate into the CNS [6] and colonize the CNS in a heterogeneous spatiotemporal pattern, with transient accumulation in selected hotspots, i.e., the cortico-striatal-amygdalar boundary, before distributing throughout the brain and developing site-specific morphological and phenotypical features [10]. As discussed above, mature microglia have a typical gene profile, with *Hexb, P2ry12*, *Tmem119*, *Sall1* and *Siglech* representing homeostatic markers [62]. However, some of these genes may vary in expression according to the developmental stage [9] or reactive states of microglia [65]. Embryonic or postnatal microglia are best visualized through *Cx3cr1*-linked reporters. Studies with transgenic mice expressing fluorescent reporters under the control of the *Cx3cr1* promotor have helped describe microglia activity during brain development, i.e., the sculpting of postnatal neuronal networks through complement C3-mediated synaptic pruning [93]. There is less information on whether microglia can affect the formation of the brain vasculature during CNS development. However, liposomal clodronate-mediated depletion of microglia has been reported to impair retinal vessel formation [17] and to alter the vascular architecture and capillary diameter via transforming growth factor beta 1 (TGFβ1)-dependent paracrine signaling [24, 26, 47].

Microglia and CAMs originate from EMPs, which differ from angiopoietic progenitors from which the brain vasculature is derived. In general, each brain cell type is derived from a specific subset of progenitor cells, following a defined spatiotemporal pattern of differentiation that spans from the yolk sac to the first postnatal weeks. However, endothelial cells can share a common erythromyeloid lineage with CNS resident macrophages. Plein et al. [80] traced EMPs in *Csf1r-Egfp* mouse embryos, reporting that at E8.5 the EMPs protrude into the vascular lumen, acquiring the typical elongated shape of the endothelial cells and expressing the VEGFR2. The EMP-derived endothelial cells are transcriptomically identical to the neighboring ones of angiopoietic origin, and lack the expression of genes of differentiated myeloid cells. Also, these cells are long-lived after their integration into the vessel wall. Thus, a new source of endothelial cells is revealed, suggesting that the embryonic blood vascular endothelium expands in a dual mechanism involving both the proliferation of pre-existing endothelial cells and the incorporation of endothelial cells derived from hematopoietic precursors [80].

## CAMs in homeostasis

Under homeostatic conditions, CAMs play an important role in maintaining the integrity of the CNS barriers as well as regulating the exchange of metabolites and antigens with the periphery [53]. It has been shown that the production of VEGF by PvMΦ impacts endothelial cell function, thereby modifying vascular permeability [46]. Furthermore, despite their limited motility, PvMΦ are potentially able to extend their processes along the perivascular space into the vascular lumen and sense molecular signals within the bloodstream [7, 35, 53]. Similarly, two-photon in vivo imaging of dmMΦ and MnMΦ suggests that these macrophages continuously scavenge their environment [94]. In fact, studies using injections of the tracer ferritin directly into the CSF have shown the phagocytic capacity of PvMΦ and epiplexus cpMΦ [15, 33, 53, 73]. Consistent with this, single-cell phenotyping of CAMs has associated them with various biological processes, such as phagocytosis, antigen presentation, and cytokine production in mice [2, 49, 71, 95, 114] and recently in humans [51, 91, 124]. Altogether, this emphasizes the pivotal role of CAMs as immune surveillants, clearing potentially harmful substances and contributing to the maintenance of CNS homeostasis.

## CAMs in neurodegenerative diseases

The study of CAMs and their impact on CNS diseases is a rapidly developing field of research. Although the precise role of CAM perturbation in CNS homeostasis is far from understood, recent studies have shed some light on their involvement in neurodegenerative and cerebrovascular diseases.

Alzheimer’s disease (AD) and Parkinson’s disease (PD) are both characterized by local microgliosis, neuronal death and the appearance of protein aggregates. While in AD, these aggregates mainly include amyloid beta (Aβ) plaques and/or tau neurofibrillary tangles [3], in PD, they are known as Lewy bodies and are primarily composed of α-synuclein (α-syn) [104]. Importantly, genome-wide association studies (GWAS) have identified genetic variants associated with an increased risk of neurodegenerative disease, many of which are significantly enriched or uniquely expressed in myeloid cells such as microglia [83].

The BBB plays a crucial role in regulating both protein diffusion and leukocyte migration, and these functions are intricately regulated. In its response to CNS inflammation, the BBB is able to regulate leukocyte migration without concurrent protein leakage, and vice versa. This underscores the precision and regulatory mechanisms governing these processes. The maintenance of BBB integrity in neurodegenerative disorders [reviewed in 116], along with the possible contribution of monocyte-derived cells in clearing plaques, remains unclear. This is due to the difficulty in distinguishing CNS resident macrophages and infiltrating myeloid populations, since in situ they show similar morphologies and phenotypes [84]. The proximity of monocyte-derived cells to Aβ plaques and their engulfment of Aβ was proposed in a recent study where *Ms4a3*^Cre^*R26*^tdTomato^ bone marrow was transplanted into 5×FAD mice, a mouse model of AD [100]. Similarly, *Flt3*^Cre^ constitutive fate mapping of bone marrow-derived cells labeled 6% of plaque-associated macrophages in APP/PS1 AD mice [123]. Conflicting earlier results showed that, in the absence of brain irradiation, using inducible *Ccr2*^*CreERT2*^ and *Cx3cr1*^*CreERT2*^ fate-mapping on a 5×FAD background, no myeloid cell infiltration is detected [89]. Similarly, in the inducible *Kit*^MerCreMer^*R26*^Eyfp^ on an APP^NL−G−F^ background, no replacement by bone marrow-derived monocytes was observed neither in microglia nor CAM populations [118]. Despite conflicting results, in vivo two-photon imaging of APP/PS1 mice reveled that patrolling Ly6C low monocytes are attracted to the luminal walls of Aβ-positive veins where they phagocyte Aβ before circulating back into the bloodstream [66].

Due to their specific anatomical location, PvMΦ and MnMΦ have been suggested to have an important role in the surveillance and control of flow dynamics of the CSF [25]. Conveniently, the injection of clodronate-containing liposomes into the cisterna magna or cerebral ventricles effectively depletes these CAM populations, albeit with significant changes in the CSF volume and osmolarity which may have collateral effects [79]. Nevertheless, such clodronate-liposome depletion studies in different mouse models of AD are associated with reduced clearance of vascular and parenchymal Aβ plaques [25, 42] (Fig. 1), potentially by PvMΦ and MnMΦ where the scavenger receptor class B type I (SR-BI) seems to play a role [111]. Yet, another depletion study showed that the expression of CD36 and Nox2 by PvMΦ and MnMΦ leads to the generation of reactive oxygen species (ROS) and consequent cerebrovascular dysfunction in *Tg2576* mice [76]. In addition, anti-Aβ immunotherapy in a mouse model of AD has been shown to activate PvMΦ, which exhibit a higher association with vascular Aβ plaques, ultimately inducing microhemorrhages and an increased monocyte infiltration [110].

A potential role of CAMs in AD has become more evident with a recent single-nucleus RNAseq study of the human brain vasculature. This study unveiled that many of the top GWAS genes were not only expressed by microglia but also by PvMΦ [124]. Interestingly, a novel scRNA-seq study in a mouse model of PD reported that CAMs, rather than microglia, play an essential role as antigen-presenting cells and mediate α-synuclein-related neuroinflammation [30, 95]. While mice lacking the antigen-presenting molecule MHCII specifically in microglia showed no differences in the recruitment of peripheral immune cells, clodronate-mediated depletion of CAMs significantly decreased infiltrating CD4 T cells and monocytes [74, 95].

Extensive research has underscored the crucial role of microglia in the human pathogenesis of neurodegenerative diseases. Immunostainings of autopsy tissues have revealed that under such conditions, microglia undergo activation and engage on the phagocytosis of protein aggregates. The advent of new single-cell technologies has allowed for a more detailed and intricate characterization of microglial states in neurodegenerative diseases, exposing a spectrum of subtypes and providing insights into their distinct gene expression profiles [74, 81]. Yet, despite the expected involvement of CAMs in human neurodegenerative conditions, data on their specific role remain scarce.

## CAMs in cerebrovascular diseases

Cerebrovascular diseases are medical conditions that influence the blood flow in the vessels supplying the brain, with ischemic stroke being the best-known and most studied pathology [14]. Lack of adequate blood supply causes brain damage and induces a pro-inflammatory immune response as well as an excessive production of ROS, which compromises BBB functional integrity. Considering their anatomical location at the CNS interfaces, it seems rational that CAMs play a role in such conditions. Consistent with this hypothesis, it has been suggested that in homeostatic conditions, Lyve-1^+^ perivascular macrophages prevent arterial stiffness in mouse aorta by influencing collagen expression by vascular smooth muscle cells [57].

Hypertension, or high blood pressure, is a significant risk factor that can lead to various vascular diseases, including ischemic stroke. It is widely recognized that microglia respond rapidly and become activated in the presence of BBB leakages. Interestingly, studies have shown that in cases of vascular injury, microglia activation occurs promptly, accumulating around vasculature even before detectable BBB damage takes place [107]. Similarly, hypertension is associated with increased PvMΦ numbers along the cerebral vasculature [58]. Interestingly, consistent with findings from AD studies, PvMΦ have been suggested to be responsible for the excessive production of ROS via Nox2 in mouse models of hypertension [28, 92]. The accumulation of ROS compromises BBB function and is associated with neurovascular and cognitive defects, which are mitigated by clodronate-mediated CAM depletion [28, 92]. Accordingly, in animal models of permanent ischemic stroke, elevated numbers of CD163^+^ CAMs have been reported near the lesion in rats [77, 86] and of Lyve1^+^ CAMs in mice [101]. These PvMΦ express VEGF, which may compromise vascular barrier function and, thereby, granulocyte recruitment [77]. Transplantation of Cx3cr1^gfp^CCR2^rfp^ bone marrow into wild-type hosts revealed that peripheral monocytes infiltrate and repopulate the perivascular spaces 4 days after ischemic stroke [86]. These results are consistent with a previous study in mice suffering from experimental autoimmune encephalomyelitis (EAE), which reported that CAM proliferation occurs alongside the infiltration of monocytes [49]. These monocyte-derived cells only transiently colonize the parenchyma but are not integrated into the microglia pool and remain functionally distinct to microglia [1, 49]. Additionally, there is clinical evidence supporting the accumulation of CD163^+^ PvMΦ in autopsied brains of patients with cerebral ischemia [45], as well as of PvMΦ-like cells in patients with brain arteriopathies [122]. Indeed, these data need to be interpreted with caution, since microglia and bone marrow-derived macrophages may upregulate CD163 and other CAM markers under certain conditions [97]. For this reason, to specifically distinguish and target each myeloid population in the context of disease, the use of fate-mapping mouse models is indispensable. The limited data on the role of human CAMs in cerebrovascular diseases underscore the importance of future research in this area.

## Emerging vascular mechanisms of myeloid cell brain infiltration

In neurodegenerative and cerebrovascular diseases, blood-borne myeloid cells may have access to the brain, where they can differentiate into mature macrophages. This process differs from that regulating myeloid cell seeding and differentiation during CNS development, due to the presence of a defined, but damaged, vascular barrier. Whether macrophages derived from such infiltrating monocytic cells are functionally equivalent to the long-term resident populations is not yet resolved.

The recruitment of myeloid cells largely depends on proteins expressed on the endothelium of postcapillary venules, including adhesion molecules controlling monocyte rolling, arrest and extravasation [reviewed in 22]. There are two emerging mechanisms to explain regulation of monocyte entry into the brain, i.e., glycocalyx-associated vascular-immune cell interactions and an endothelial basement membrane (laminin)-driven infiltration and differentiation into macrophages.

Two subsets of monocytes have been identified based on their ability to extravasate (Ly6C^high^CCR2^high^CXCR1^low^) or to patrol the vessels (Ly6C^low^CCR2^low^CXCR1^high^) [31]. These phenotypes represent the extremes of a range of phenotypes that are defined by environmental factors that also control bone marrow-derived monocyte migration, differentiation, and tissue entry. Brain resident myeloid cell replacement by monocytes during homeostasis is limited and restricted to dura and choroid plexus macrophages only [80]. By contrast, tissue-resident macrophages located in other organs are more frequently replenished [120] through a mechanism involving both the endothelial cells and their underlying basement membrane. This latter structure has been recently reported as a critical cue for monocyte differentiation into macrophages in the intestine, with a specific role of laminin proteins [56]. The two main endothelial laminins, laminin 411 and 511, are constituents of vascular basement membranes of arteries, arterioles and capillaries, with progressively less laminin 511 in postcapillary venules, venules and veins. Postcapillary venules which have low or no laminin 511 are the preferred sites for leukocyte and monocyte extravasation in both the brain and peripheral organs [56, 102, 103, 117]. Laminin 511 together with the endothelium provide a cue for monocyte differentiation, as observed in the intestine of endothelial cell-specific laminin 511 knockout mice (Tek-cre:*Lama5*^*−/−*^), which show reduced proportions of mature macrophages—identified as Ly6C^low^MHCII^high^—compared to wild type or laminin 411 depleted (*Lama4*^*−/−*^) mice despite higher numbers of infiltrating Ly6C^high^/MHCII^low^ monocytes. By contrast, *Lama4*^*−/−*^ mice which have a high expression of laminin 511 have less extravasation of immature (Ly6C^high^MHCII^low^) and maturing (LY6C^high^MHCII^high^) monocytes but higher proportions of differentiated macrophages [56]. The data suggest that laminin 411 in the postcapillary venule basement membrane supports immature monocyte extravasation, while laminin 511 together with the endothelium promotes their differentiation into macrophages. In the brain, the differential expression of laminins 411 and 511 in endothelial basement membranes have also been shown to modulate the pathogenicity of infiltrating T cells during neuroinflammation [127]. Taken together these data suggest that laminins 411 and 511 may also control monocyte recruitment and differentiation during neuroinflammation.

As discussed above, the endothelial glycocalyx—a negatively charged, carbohydrate-rich structure—is a major regulator of immune cell trafficking. Being exposed on the luminal surface of the endothelium, glycoproteins are accessible to circulating cells and proteins. In physiological conditions, the glycocalyx contributes to the barrier function of the BBB, together with the endothelium and the extravascular cellular and basement membrane components. That the glycocalyx forms a CNS barrier was demonstrated by Kutuzov et al. [55] who intravenously injected fluor-conjugated wheat-germ agglutin into mice to selectively label N-acetylglucosamine and sialic acid, components of the glycocalyx. In addition, mice were injected with sulforhodamine 101 to label the astrocytic endfeet, and 40 or 150 kDa MWt fluorescein isothiocyanate (FITC)-dextran. Diffusion of FITC-dextran across the BBB was followed by live in vivo two-photon microscopy, revealing accumulation in the glycocalyx which was 44% of the signal intensity in the blood. By modeling a partition coefficient, the authors concluded that the glycocalyx is one of the three structures, including the endothelium and the extravascular components, forming a sequence of diffusional constraints that was termed the tripartite BBB [55]. It is, therefore, not surprising that disruption of the glycocalyx, as occurs in inflammation and disease conditions, compromises BBB function. Moreover, proteins shed from the glycocalyx provide soluble damage-associated molecular patterns (DAMPs), further enhancing inflammation and immune cell recruitment [69]. In patients with acute heart failure syndromes, circulating levels of heparan sulfate proteoglycan degradation products derived from the glycocalyx were found to be elevated compared to aged-matched healthy controls and were associated with increased CD14 levels, a marker of monocyte activation [38]. Evidence of monocyte activation by products of glycocalyx disruption was reported in lipopolysaccharide (LPS)-treated mice and linked to the activation of Toll-like receptor 4 (TLR4) [29]. Heparan sulfate-rich vascular domains are particularly involved in the regulation of thrombo-inflammatory events, presenting binding sites for different growth factors, cytokines and chemokines [69]. Chemokines, like the monocyte chemoattractant protein (MCP) 1, and the macrophage inflammatory peptides (MIP) 1α and β interact with the glycocalyx to generate a local concentration of chemokines (‘chemokine-cloud’), facilitating leukocyte activation and amplifying pro-inflammatory signals [36, 54]. At present, how the glycocalyx is modified in different diseased brain conditions, and how this may affect monocyte recruitment is not fully understood. Glycans shed from the injured endothelium could potentially bind and modulate inflammatory molecules systemically [108], but also the modification of endothelial cell surface-exposed sugars may direct monocyte interaction with the endothelium, as recently hypothesized in a murine model of ischemic stroke [75].

## Conclusions

Although the origin of CAMs has been recently elucidated in mice, the specific *niche* factors that facilitate their proper development, migration, engraftment, and long-term maintenance in the distinct CNS compartments remain largely unknown. Data suggest that the brain vasculature and the extracellular matrix constituting the microenvironment are likely to provide the cues to maintain CAM phenotype and functions, once they are established within their *niches*. Encouragingly, recent studies have begun elucidating the role of CAMs in CNS development, homeostasis and perturbations. Nevertheless, further studies are needed to fully comprehend the intricate networks through which CAMs contribute to disease pathogenesis.

The use of animal models that specifically target CAMs as well as the development of new state-of-the-art techniques that allow precise characterization of both CAMs and their interaction with other brain resident cells holds great promise for understanding CAM functionality and their potential as therapeutic targets. Given the pivotal role of these cells in neurodegenerative, cerebrovascular and neuroinflammatory diseases, more studies that specifically interrogate CAMs in these pathogenesis are needed. While new technologies have facilitated the identification of markers distinguishing microglia and CAMs, aiding in the dissection of their distinct functionalities, a deeper characterization of individual CAM populations will enable the study of their precise *niche* functions in both homeostasis and CNS perturbation. This is of special interest in human studies, where the complexity of CAM subsets is just starting to be unveiled. Moreover, a more comprehensive understanding of myeloid cell engraftment in the CNS parenchyma and interfaces, particularly now that it has been suggested in humans, opens up promising avenues for potential CAM and microglia replacement therapies.
